# Demystifying Clinical Appropriateness in Virtual Care and What Is Ahead for Pay Parity: Proceedings of the 3rd Annual Mass General Brigham Virtual Care Symposium

**DOI:** 10.1089/tmr.2023.0015

**Published:** 2023-04-27

**Authors:** Lee H. Schwamm

**Affiliations:** Department of Neurology, Massachusetts General Hospital, Mass General Brigham Health System and Harvard Medical School, Boston, Massachusetts, USA.

Although virtual care delivery has existed in some form for over two decades,^1^ the COVID-19 pandemic thrust it into the national spotlight. Each year since 2020, a national group of experts in virtual care have gathered to address the most pressing topics of the day (see https://www.virtualcareconsensus.com for recordings of prior symposia**)**. These experts were selected for their long history of virtual care and deep implementation experience within academic health systems across the country, experience that enabled them to lead the way forward nationally in the adoption and refinement of virtual care delivery throughout the massive COVID-19-driven expansion.

We began by rethinking curriculum, competency, and culture in the virtual care era in 2020, including defining a framework for assessing competency for training in virtual care, and addressing challenges, workflows, strategies, and best practices in virtual care-enabled education.^2^ We then pivoted in 2021 to assessing the frameworks for measuring and ensuring quality in virtual care delivery, defining the guiding principles necessary for the future of virtual care measurement, best practices deployed to measure the quality of virtual care and how they compare and align with in-person frameworks.^3^ Particularly important was how rapidly increased adoption of virtual care impacted patient access and experience, and provide examples of challenges, pitfalls, and actual frameworks that have been put into place.

This year's symposium focused on the postexpansion phase of sustainability, namely looking at how best to define clinical appropriateness within virtual care delivery, and how the payment system will play a critical role in the future of virtual care. The accompanying articles underscore the importance of considering virtual care within the broader context of digital patient experience, with a critical emphasis on digital health equity. COVID-19 highlighted the stark contrasts in access to care, mortality, and despair that was disproportionately experienced by people of color, those with limited English or digital proficiency, and other unfavorable social determinants of health.

The continued and expanding mental health crisis that has followed in the wake of the COVID-19 pandemic is not unlike that of the tsunami that follows the earthquake, often with deadlier consequences, and the symposium offers suggestions as to how to dismantle barriers and strengthen the systems of care. To be effective and good stewards of our large but limited health care resources, we must strive to deliver the highest value health care possible.

That requires that we first build a framework for determining which care delivery channels and methods are clinically appropriate for any given health care encounter, and guide patients to the most safe, timely, effective, equitable, efficient, and patient-centered of these options. Only then can we embrace alternative payment models, value-based care solutions, and innovations in fee-for-service reimbursement contracts that equitably deliver the highest quality and the lowest cost.

Throughout the accompanying articles, we have chosen to standardize our terminology for the benefit of the reader, though many of the synonymous terms are used interchangeably in the literature. We have chosen the terms “social determinants of health” and “health inequity” to describe the many societal factors that lead to unequal distribution of resources, access to care, and health outcomes, recognizing that in our complex health care system while virtual care solutions may bridge a care divide for patients in some scenarios, it can also increase that divide for others.

The term “virtual care” is used to describe the delivery of digitally enabled care that has been referred to by terms such as “telemedicine, telehealth, digital care, and digital health” (https://idhi.uams.edu/rtec/wp-content/uploads/sites/4/2022/05/Telehealth-Definitions-Paper-06MAY2022-1.pdf). This includes both synchronous and asynchronous means of communicating with and monitoring the health of patients, both during and between encounters.

On behalf of the symposium organizers, I hope that this collection of articles serves as a pragmatic blueprint for those readers interested in implementing or critically examining their own virtual care delivery models.
